# Supplementary material for: Mapping the evolution of fertility support policies in China: A content and instrumental analysis
Source: PLoS One. 2025 Oct 9;20(10):e0332137. doi: 10.1371/journal.pone.0332137 (PMC12510515; doi:10.1371/journal.pone.0332137)
Supplement: S1 Appendix — (ZIP) [file pone.0332137.s001.zip › S1 Appendix. 226 original policy documents/214-第十二届全国人民代表大会第四次会议关于2015年国民经济和社会发展计划执行情况与2016年国民经济和社会发展计划的决议(FBM-CLI-1-266529).doc]

第十二届全国人民代表大会第四次会议关于2015年国民经济和社会发展计划执行情况与2016年国民经济和社会发展计划的决议

**发布部门：** [全国人民代表大会](https://www.pkulaw.com/law/chl?Aggs.IssueDepartment=101)

**发布日期：**2016.03.16

**实施日期：**2016.03.16

**时效性：** [现行有效](https://www.pkulaw.com/law/chl?Aggs.TimelinessDic=01)

**效力级别：** [工作文件](https://www.pkulaw.com/law/chl?Aggs.EffectivenessDic=XA0105)

**法规类别：** [人大议事](https://www.pkulaw.com/law/chl?Aggs.Category=00106) [营商环境优化](https://dobusiness.pkulaw.com/)

第十二届全国人民代表大会第四次会议关于2015年国民经济和社会发展计划执行情况与2016年国民经济和社会发展计划的决议
（2016年3月16日第十二届全国人民代表大会第四次会议通过）

　　第十二届全国人民代表大会第四次会议审查了国务院提出的《关于2015年国民经济和社会发展计划执行情况与2016年国民经济和社会发展计划草案的报告》及2016年国民经济和社会发展计划草案，同意全国人民代表大会财政经济委员会的审查结果报告。会议决定，批准《关于2015年国民经济和社会发展计划执行情况与2016年国民经济和社会发展计划草案的报告》，批准2016年国民经济和社会发展计划。

关于2015年国民经济和社会发展计划执行情况与2016年国民经济和社会发展计划草案的报告
--2016年3月5日在十二届全国人民代表大会第四次会议上
国家发展和改革委员会

各位代表：
　　受国务院委托，现将2015年国民经济和社会发展计划执行情况与2016年国民经济和社会发展计划草案提请十二届全国人大四次会议审议，并请全国政协各位委员提出意见。
　　**一、**2015年国民经济和社会发展计划执行情况
　　去年以来，国际经济环境错综复杂，国内经济下行压力加大，各类风险挑战明显增多。各地区各部门在党中央国务院的正确领导下，坚持稳中求进工作总基调，主动适应经济发展新常态，认真执行十二届全国人大三次会议审议批准的2015年国民经济和社会发展计划，落实全国人大财政经济委员会的审查意见，扎实做好各方面工作，经济运行总体平稳，稳中有进，稳中有好，计划执行情况总体是好的。2015年主要目标任务的完成，标志着“十二五”规划胜利收官，我国站在了更高的发展水平上。
　　（一）进一步完善宏观经济政策，经济保持中高速增长。创新宏观调控思路和方式，在区间调控基础上加强定向调控、相机调控，内需拉动力增强，出口占国际市场份额继续提高。国内生产总值达到67.67万亿元，增长6.9%，实现年度预期目标。
　　一是投资关键作用有效发挥。围绕补短板、调结构，加大有效投资力度。把重大工程建设作为促进投资增长的“牛鼻子”，优化中央预算内投资结构，设立和投放专项建设基金，多措并举鼓励和吸引社会投资，11大类重大投资工程包建设有效推进。全社会固定资产投资增长9.8%，民间投资占固定资产投资（不含农户）比重提高到64.2%。

　　二是消费潜力继续释放。扎实推进6大领域消费工程，实现投资3万亿元，带动相关消费2.8万亿元。旅游、网购、新能源汽车等消费新增长点不断培育壮大，社会消费品零售总额增长10.7%。

　　三是经济结构进一步优化。消费超投资、三产超二产的特征更加明显，消费对经济增长的贡献率达到66.4%；第三产业增加值占国内生产总值的比重首次过半，达到50.5%。城乡区域协调协同性增强，新型城镇化积极推进，常住人口城镇化率56.1%。
　　四是就业形势总体稳定。新一轮促进就业创业措施出台，创业创新对就业的带动作用增强。城镇新增就业1312万人，年末城镇登记失业率4.05%。
　　五是价格总水平温和上涨。居民消费价格上涨1.4%。价格收费监管和反垄断执法深入开展，依法查处价格违法案件3.42万件，实施经济制裁104.77亿元，减轻企业负担约89亿元。
　　（二）改革开放向纵深推进，市场活力和社会创造力不断释放。简政放权、放管结合、优化服务深入推进，重要领域改革取得积极进展，开放型经济新体制加快构建。
　　一是行政审批制度改革不断深化。取消和下放311项行政审批事项，全部取消非行政许可审批，取消214项中央指定地方实施的行政审批事项，取消123项职业资格许可和认定事项。取消国务院部门行政审批中70%的中介服务事项。取消属于企业经营自主权范围的18项核准前置手续。简政放权三张清单、四大平台初步建立。“三证合一”、“一照一码”全面实施，新登记注册企业443.9万户，增长21.6%。[不动产登记暂行条例](https://www.pkulaw.com/chl/b44f14dd582024e1bdfb.html)正式实施。

　　二是财税金融改革扎实推进。中央对地方转移支付制度进一步完善，税收征管体制改革启动，资源税从价计征范围扩大，营改增改革稳步实施。促进普惠金融、互联网金融、民营银行发展等政策文件发布实施，取消存款利率浮动上限，利率管制基本放开。存款保险制度正式实施。完善人民币汇率中间价形成机制，人民币加入国际货币基金组织特别提款权（SDR）货币篮子。政策性、开发性金融机构改革取得新进展。采取有效措施防范系统性金融风险。
　　三是投融资体制持续创新。开展政府和社会资本合作（PPP），通过特许经营、投资补助等多种方式，鼓励引导社会资本参与基础设施和公用事业建设运营。调整和完善固定资产投资项目资本金比例制度。适当调整和放宽企业债券发行条件，推出城市停车场、地下综合管廊、战略性新兴产业、养老产业、双创孵化、配电网建设改造等6个专项债券和项目收益债券、绿色债券创新品种。
　　四是价格改革力度加大。印发实施[推进价格机制改革的若干意见](https://www.pkulaw.com/chl/3f0107919d92e26ebdfb.html)。放开、下放近40项商品和服务价格。修订政府定价目录，中央、地方具体定价项目分别减少80%和55%。降低燃煤发电上网电价和工商业用电价格，大幅降低非居民用天然气门站价格。健全节能环保价格政策。继续清理规范进出口环节收费。

　　五是国有企业和重点行业改革稳步展开。10项国有企业改革试点启动实施。国资委履行出资人职责的中央企业从112家减少到106家。新一轮电力体制改革启动。石油天然气勘查开发体制改革开始试点。国有林场和国有林区、水利、供销社、农垦改革有序推进。

　　六是社会领域改革继续深化。城乡义务教育经费保障机制进一步完善。机关事业单位养老保险制度改革启动。在100个城市开展公立医院综合改革试点，县级公立医院综合改革全面推开，城乡居民大病保险覆盖所有基本医保参保人，分级诊疗制度积极推进。临时救助制度全面实施。降低失业、工伤、生育保险费率，每年可减少企业缴费600多亿元。中央和国家机关公务用车制度改革全面完成，中央企事业单位公务用车制度改革启动实施，地方党政机关公务用车制度改革深入推进。行业协会商会与行政机关脱钩试点全面推开。青海等9省市开展国家公园体制试点。
　　七是对外开放新格局加快形成。[构建开放型经济新体制的若干意见](https://www.pkulaw.com/chl/761225a1c27c2543bdfb.html)发布实施。推进共建“一带一路”的愿景与行动正式发布，“六廊六路多国多港”建设取得积极进展。对“一带一路”相关国家直接投资148亿美元，增长18.2%。国际产能和装备制造合作指导意见发布实施，铁路、核电等走出去实现突破。亚洲基础设施投资银行正式成立，丝路基金启动运行。[支持沿边重点地区开发开放若干政策措施的意见](https://www.pkulaw.com/chl/85fe40c8461466bdbdfb.html)出台。修订[外商投资产业指导目录](https://www.pkulaw.com/chl/4804e2412f5e1853bdfb.html)，外商投资限制类条目减少50%，有股比要求的条目减少40%，95%以上项目实现备案管理。外债管理由审批制改为备案登记制。新设立广东、天津、福建3个自贸试验区，上海自贸试验区扩展区域。签署中韩、中澳自贸协定和中国－东盟自贸区升级议定书。非金融类外商直接投资1263亿美元，增长5.6%，其中服务业利用外资比重超过60%。非金融类境外直接投资1180亿美元，增长14.7%。

　　（三）坚持创新驱动发展，新产业新业态加快形成。出台[深化体制机制改革加快实施创新驱动发展战略的若干意见](https://www.pkulaw.com/chl/b45d37e3f1ff5d80bdfb.html)，推进全面创新改革试验，大力推动创新和产业升级。
　　一是大众创业、万众创新蓬勃发展。大力推进大众创业、万众创新若干政策措施的意见出台实施。“互联网+”行动计划启动，大数据、电子商务等领域新业态不断涌现。众创众包众扶众筹支撑平台加快发展，全国各类众创空间超过2300家，技术转移示范机构达453家，技术交易总额接近1万亿元。国家新兴产业创业投资引导基金设立。中小企业公共服务平台在26个省实现互联互通。首届全国双创活动周成功举办。

　　二是传统产业转型升级步伐加快。“中国制造2025”和增强制造业核心竞争力三年行动计划启动实施，在增强制造业核心竞争力、产业振兴和技术改造等领域推进一批重大工程。具有自主知识产权的中国标准动车组成功下线，一批现代农业机械高端产品取得突破，成功组建国家机器人检测与评定中心，自主品牌工业机器人示范应用顺利推进。先进制造产业投资基金设立，首期基金规模200亿元，有效吸引了社会资本参与。
　　三是坚定不移化解产能严重过剩矛盾。清理整顿违规项目，严禁备案和建设产能严重过剩行业新增产能项目，产能扩张势头得到遏制。全年淘汰炼铁产能1300万吨、炼钢1700万吨、水泥3800万吨、电解铝30万吨、平板玻璃1100万重量箱以上。煤炭行业脱困深入推进。
　　四是战略性新兴产业取得新进展。国家民用空间基础设施建设全面启动，我国自主研制的C919大型客机总装下线，ARJ21新型支线飞机实现首架交付。北斗卫星导航系统广泛应用，云计算、基因检测、新能源汽车、机器人、移动互联网等行业快速发展，集成电路、海洋工程装备、医疗器械等行业向高端迈进。
　　五是现代服务业加快创新发展。[加快发展生活性服务业促进消费结构升级的指导意见](https://www.pkulaw.com/chl/12d410c7782a4a0abdfb.html)出台，服务业市场准入进一步放开，综合改革试点扎实推进，跨境电子商务综合试验区进展顺利。第三产业增加值增长8.3%。
　　六是基础设施网络更加完善。综合交通体系建设全面推进，网络总里程达到494万公里。能源供应能力增强，消费结构优化，非化石能源消费比重上升到12%，煤炭消费比重下降到64%。“宽带中国”战略深入推进，新一代信息基础设施日趋完善。

　　（四）推动城乡区域协调发展，新的增长点增长极增长带加快形成。“三大战略”、“四大板块”、新型城镇化积极推进，新的发展空间不断拓展。
　　一是“三大战略”正在从蓝图向现实转化。“一带一路”建设开局良好。京津冀协同发展稳步前行，北京非首都功能疏解有序开展，交通、生态、产业三个重点领域率先突破加快推进。长江经济带发展深入推进，绿色生态廊道加快构建，综合交通体系逐步完善。

　　二是“四大板块”发展更趋协调。新一轮西部大开发战略加快落实，采取有效举措积极应对东北地区经济下行压力，中部地区综合交通枢纽和生态经济区建设步伐加快，东部地区在改革创新、转型升级、提质增效方面继续发挥示范作用。国家级新区建设发展势头良好。

　　三是新型城镇化积极稳妥推进。“一融双新”工程进展顺利，27个省区市和新疆生产建设兵团出台户籍制度改革实施意见，[居住证暂行条例](https://www.pkulaw.com/chl/73486fc333ab2ac2bdfb.html)印发实施。新型城市建设和新生中小城市培育有序推进。长三角、成渝、哈长等城市群规划编制完成。国家新型城镇化综合试点扩大到2个省和135个城市（镇）。市县“多规合一”改革取得积极成效。
　　（五）加快转变农业发展方式，农业现代化扎实推进。农业和农村经济平稳发展，粮食产量实现“十二连增”。
　　一是农业支持政策创新完善。“三农”投入继续加大，中央预算内投资用于“三农”的比重连续5年超过50%。全国涉农资金专项整治行动圆满完成，省级涉农资金整合优化试点启动实施。粮食最低收购价和临时收储政策逐步完善，新疆棉花、东北和内蒙古大豆目标价格改革试点进展顺利。粮棉油糖化肥等重要商品市场调控加强。
　　二是农业农村基础设施建设继续强化。重大水利工程建设加快推进。大中型灌区节水改造和新增1000亿斤粮食生产能力规划田间工程加快实施，现代种业基础设施建设得到加强，粮食收储仓容建设力度加大。农村水电路气房等基础设施建设深入推进，城乡基础设施和公共服务设施互联互通、共建共享步伐加快。农业面源污染治理、东北黑土地保护利用、已垦草原治理试点启动，耕地重金属污染治理、地下水超采治理试点积极推进。

　　三是农业结构调整迈出新步伐。粮食产量6.21亿吨，肉蛋奶、果蔬茶、水产品等重要农产品丰产丰收。畜禽养殖规模化率提高到54%，粮改饲、粮豆轮作试点全面启动。农作物耕种收综合机械化水平达到63%，农业科技进步贡献率56%。
　　四是农村改革稳步推进。农村土地征收、集体经营性建设用地入市和宅基地制度改革试点在33个县级行政区域实施，农村承包土地经营权和农民住房财产权抵押贷款试点在278个县级行政区域实施。农村集体资产股份权能改革试点有序推进。家庭农场、农民合作社、产业化龙头企业等新型农业经营主体近250万家。
　　（六）生态文明建设力度加大，节能环保目标超额完成。单位国内生产总值能耗和二氧化碳排放量分别下降5.6%和6%以上，二氧化硫、化学需氧量、氨氮、氮氧化物排放量分别下降5.8%、3.1%、3.6%和10.9%，万元工业增加值用水量下降3.9%。
　　一是生态文明建设持续加强。[加快推进生态文明建设的意见](https://www.pkulaw.com/chl/7ca3e285623035afbdfb.html)和[生态文明体制改革总体方案](https://www.pkulaw.com/chl/442c015bf83fd9d1bdfb.html)发布实施，生态文明先行示范区建设进展顺利。主体功能区环境政策、全国海洋主体功能区规划、全国生态功能区划印发实施。扩大新一轮退耕还林还草工程实施范围，完成造林面积632万公顷，全国草原综合植被盖度达54%。石漠化治理、湿地保护得到加强，新增水土流失综合治理面积540万公顷。
　　二是节能减排深入开展。在大气污染治理重点城市实行煤炭消费总量控制，煤电节能减排升级改造行动计划全面实施。环境污染第三方治理、再制造产品“以旧换再”和生产者责任延伸制度等试点示范有序开展。绿色建筑积极推进。农作物秸秆综合利用得到加强。
　　三是污染治理全力推进。首批实施新环境空气质量标准的74个城市细颗粒物（PM2.5）平均浓度下降14.1%。重点流域水污染防治和湖泊生态环境保护工程力度加大，地表水达到或好于Ⅲ类水体比例提高到66%。启动土壤污染治理与修复试点示范项目，推进污染场地环境监管试点。
　　四是应对气候变化取得新进展。启动第一批低碳城（镇）试点。在北京、天津、上海、重庆、广东、湖北、深圳等7个地方推进碳排放权交易试点。建设性参与气候变化巴黎大会，提出全球气候治理中国方案，推动达成巴黎协定。
　　（七）更加注重保障和改善民生，民生福祉持续改善。多措并举增投入、补短板、兜底线，扎牢民生保障网。
　　一是精准扶贫精准脱贫步伐加快。易地扶贫搬迁、产业扶贫、转移就业、教育扶贫、健康扶贫等扶贫工程启动实施。全年农村贫困人口减少1442万人。对革命老区开发建设、赣南等原中央苏区振兴发展的支持力度明显加大。加强中央支持和对口支援，进一步促进新疆、西藏和四川、云南、甘肃、青海四省藏区经济社会发展和长治久安。
　　二是居民收入稳定增长。全国居民人均可支配收入实际增长7.4%，其中农村居民人均纯收入突破万元大关，实际增长7.5%，增幅连续第6年高于经济增速和城镇居民收入增幅。城乡居民收入比下降到2.73:1。
　　三是社会保障水平稳步提高。基本养老保险参保人数达到8.58亿人，基本医疗保险参保率稳定在95%以上。企业退休人员养老金水平继续提高，城乡居民基础养老金最低标准由每人每月55元提高到70元。城乡居民医保财政补助标准由每人每年320元提高到380元。特困人员供养制度开始规范建立，20个省市建立了困难残疾人生活补贴和重度残疾人护理补贴制度，800多万残疾人受益。
　　四是公共服务供给水平进一步提升。贫困地区义务教育薄弱学校办学条件持续改善，乡村教师支持计划启动实施，农村贫困地区学生进入重点大学渠道进一步畅通。九年义务教育巩固率、高中阶段教育毛入学率分别达到93%和87%。基层医疗服务体系、重大疾病防治体系、全科医生培养基地建设加强，人均基本公共卫生服务经费达到40元，服务项目扩大到12类。国家基本公共文化服务指导标准发布实施，地市级公共文化服务设施建设支持力度加大。体育产业创新发展取得积极进展。

　　五是保障性安居工程建设顺利推进。加大中央预算内投资和中央财政专项资金支持力度，安排专项建设基金和企业债券融资，支持棚户区改造等保障性安居工程。全年城镇保障性安居工程新开工783万套，基本建成772万套。
　　从计划指标运行情况看，经济增长、价格总水平、国际收支平衡、就业等总量指标保持在合理区间，一些反映经济结构和质量的指标进一步改善，社会发展和民生保障类指标继续向好，资源节约利用和环境保护类指标完成情况较好，计划指标的完成情况总体是好的。
　　18个约束性指标全部完成。41个预期性指标中，35个运行情况符合或好于预期，5个指标运行值与预期值存在差距，1个由于统计口径变化数据不可比。需要说明的是，预期性指标的计划目标不是指令性的，也不是预测值，而是国家期望的发展目标，体现预期和政策导向，实际运行情况是市场行为的客观结果，可能高于预期目标，也可能低于预期目标。部分指标运行值与预期目标存在差距，有以下几种情况。一是为体现宏观调控内需导向，社会消费品零售总额和全社会固定资产投资指标定得比预测值稍高一些，是全年的努力目标，实际运行结果与预期目标会有一定差距。社会消费品零售总额指标受输入性通缩以及国内部分工业品价格下跌、农产品价格涨幅有限等影响，实际增幅基本符合预期，但名义增长则低于年初确定的预期目标。全社会固定资产投资指标，主要是在国际市场低迷、内需不足的情况下，部分制造业行业出现产能严重过剩，加上三四线城市房地产库存高企以及投资品价格降幅持续扩大等因素影响，全年增长也低于年初确定的预期目标。二是有的指标低于预期存在特殊因素。2015年经济下行压力加大、企业利润持续下滑，制约了研发投入，加上2014年研究与试验发展经费支出与国内生产总值之比的统计数据，由年初的2.09%修正为年末的2.05%，使得2015年预期目标未能实现。三是国际经济环境变化导致有的指标全年数值低于预期目标。受全球贸易放缓、国际能源价格大幅下跌影响，进出口总额指标增速低于预期目标。国际大宗商品价格大幅下跌，加之国内市场需求不旺，导致天然气产量指标未能达到全年预期目标。此外，由于主管部门调整了户籍人口统计口径，户籍人口城镇化率指标2015年实际数与计划数不可比。
　　总之，在国内外形势错综复杂的情况下，我国经济社会发展取得的成就来之不易。这是党中央国务院正确领导的结果，是各地区各部门共同努力的结果，是全国各族人民团结奋斗的结果。经过五年的努力，“十二五”规划《纲要》主要指标顺利完成。
　　同时，我们也清醒地认识到，世界经济继续深度调整、分化明显，全球经济、国际贸易双双低速增长，地缘政治风险上升，复苏基础仍相当脆弱，外部环境的不稳定不确定因素增加。国内经济处于“三期叠加”阶段，经济持续下行带来的不利影响和长期积累的深层次矛盾特别是结构性矛盾进一步显现，发展面临的形势可能更加错综复杂，困难可能会更大。一是经济下行压力加大。需求放缓趋势仍在延续。外需总体疲弱，外贸形势更加严峻复杂。受工业品价格持续低迷、劳动力等要素成本上升影响，投资特别是制造业投资增长乏力。随着经济下行压力逐步向就业、收入领域传导，对居民消费的影响可能显现。二是实体经济困难继续加重。受需求不足和综合成本上升的双重挤压，部分行业企业盈利能力下降、亏损增加，困难企业增多。一些行业和企业出现裁员或隐性失业情况。三是结构性矛盾进一步凸显。有效需求乏力和有效供给不足并存，新旧动力转换不均衡。供给侧结构性矛盾更加突出，供给体系调整滞后，有效供给不能适应需求总量和结构变化，结构性产能过剩依然比较严重。四是生态环境问题依然突出。大气污染形势严峻，严重雾霾天气在一些城市和地区时有发生。水环境质量偏低，一些地方地下水严重超采。一些地区土壤污染治理任务艰巨。环境基础设施建设滞后。五是各方面风险因素积聚交织。财政收入增速放缓，收支矛盾突出，地方债务存在局部风险隐患。银行业不良贷款余额和比例“双升”，企业负债率上升，非法集资多发，潜在金融风险仍在积聚。与此同时，安全生产和民生等领域也出现一些新问题。我们要高度重视，充分估计经济下行带来的困难和挑战，增强忧患意识，坚持底线思维，既保持战略定力，又及时做好预警预案预控，更加有效应对，认真加以解决。

　　**二、**2016年经济社会发展要引领经济新常态、贯彻发展新理念
　　2016年是全面建成小康社会决胜阶段的开局之年，是推进结构性改革的攻坚之年。做好2016年经济工作，对于全面建成小康社会，实现“十三五”奋斗目标，具有十分重要的意义。
　　（一）总体要求
　　全面贯彻党的十八大和十八届三中、四中、五中全会精神，以邓小平理论、“三个代表”重要思想、科学发展观为指导，深入贯彻习近平总书记系列重要讲话精神，加强和改善党对经济工作的领导，统筹国内国际两个大局，按照“五位一体”总体布局和“四个全面”战略布局，牢固树立和贯彻落实创新、协调、绿色、开放、共享的发展理念，适应经济发展新常态，坚持改革开放，坚持稳中求进工作总基调，坚持稳增长、调结构、惠民生、防风险，实行宏观政策要稳、产业政策要准、微观政策要活、改革政策要实、社会政策要托底的总体思路，保持经济运行在合理区间，战略上坚持持久战，战术上打好歼灭战，着力加强结构性改革，在适度扩大总需求的同时，去产能、去库存、去杠杆、降成本、补短板，提高供给体系质量和效率，提高投资有效性，加快培育新的发展动能，改造提升传统比较优势，增强持续增长动力，推动我国社会生产力水平整体改善，努力实现“十三五”时期经济社会发展的良好开局。
　　（二）主要预期目标
　　按照上述总体要求，综合考虑需要和可能，提出2016年经济社会发展的主要预期目标：
　　--保持经济平稳健康发展。国内生产总值预期增长6.5%－7%。主要考虑：一是为“十三五”全面建成小康社会打下好的基础。到2020年全面建成小康社会，“十三五”期间需要经济年均增长6.5%以上，如果今年增速低于6.5%，后几年就需要提高增速。为避免压力推后，今年预期目标设定为6.5%－7%，可以争取主动。二是更加有效地促进就业。从经济增长与就业关系看，6.5%－7%的经济增长能够带动1000万人以上的城镇新增就业。三是更加积极地引导预期增强信心。6.5%－7%的增长预期目标，扩大了可接受的经济增速弹性范围，与我国经济增长潜力和市场预期相吻合，能够起到提振信心的积极作用。同时也要看到，我国发展环境更加错综复杂，经济增速仍在探底之中，实现这一目标还需要更大力度的政策和更加艰苦的努力。
　　--经济提质增效升级取得新进展。投资、消费保持稳定增长，消费对经济增长的贡献率稳步提高。农业基础进一步加强，工业转型升级取得积极成效，战略性新兴产业加快发展，服务业占国内生产总值比重继续提高。科技进步对经济发展支撑作用增强，研究与试验发展经费与国内生产总值之比稳定提高。单位国内生产总值能耗和二氧化碳排放量分别下降3.4%以上和3.9%以上，万元国内生产总值用水量下降5.1%，化学需氧量、氨氮、二氧化硫、氮氧化物排放量分别下降2%、2%、3%、3%，细颗粒物（PM2.5）未达标地级及以上城市浓度降低3%，地级及以上城市空气质量优良天数比率达到77%，地表水达到或好于Ⅲ类水体比例66.5%，劣V类水体比例控制在9.2%以内。
　　--价格总水平基本稳定。居民消费价格涨幅预期目标为3%左右。主要考虑：一是翘尾因素略高于上年。据测算，今年翘尾因素的影响比上年的0.5%有所上升。二是存在一些新涨价因素。需求偏弱决定了物价不具备大幅反弹的基础，但考虑到人工等成本上升还会推动服务价格上涨，少数农产品存在周期性波动的可能，加上推进价格改革的影响，预计新涨价因素也会有所提升。三是积极引导市场预期。为改善通缩预期，将价格总水平预期目标适当定高一些。
　　--民生福祉继续增加。城镇新增就业1000万人以上，城镇登记失业率控制在4.5%以内，既突出保就业的底线，又为化解过剩产能、推进企业兼并重组、应对隐性失业显性化留出一定余地。居民收入增长与经济增长同步，城乡收入差距进一步缩小，农村贫困人口减少1000万人以上。户籍人口城镇化率提高1.3个百分点以上。社会保障制度继续完善，社会保险统筹层次进一步提高，覆盖面继续扩大，保障水平适度提高。教育、卫生、文化等基本公共服务覆盖范围和均等化水平不断提高。
　　--国际收支保持基本平衡。货物贸易出口增速高于世界贸易增长水平，进口规模扩大，服务贸易占外贸总额比重继续提高。利用外资保持增长，对外直接投资较快增长。
　　（三）宏观经济政策取向
　　中央经济工作会议对适应引领经济发展新常态提出了明确要求，特别是对新常态怎么看、新常态下怎么干，强调了“三个必须”和“十个更加注重”。“十三五”规划《建议》提出了创新、协调、绿色、开放、共享新发展理念，将引领“十三五”乃至更长时期的经济社会发展。实现2016年经济社会发展目标，要围绕引领经济新常态、贯彻发展新理念，在适度扩大总需求的同时，着力加强供给侧结构性改革，落实好宏观政策要稳、产业政策要准、微观政策要活、改革政策要实、社会政策要托底的总体思路，做到相互配合、协调发力。
　　--宏观政策要稳。稳定和完善宏观经济政策，加强区间调控、定向调控、相机调控，加大对实体经济支持力度，确保经济运行在合理区间。
　　一方面，积极的财政政策要加大力度。今年拟安排全国财政赤字2.18万亿元，比去年增加5600亿元，赤字率3%，其中，中央财政赤字1.4万亿元，地方财政赤字7800亿元。一是阶段性提高财政赤字率，在适当增加财政支出和中央预算内投资的同时，主要用于弥补减税降费带来的财政减收，保障政府应该承担的支出责任。二是优化财政支出结构，创新财政支出方式，盘活财政资金存量，该保的保住，该减的减下来。三是适当增加地方专项债券发行，主要用于支持土地储备、交通、水利及城镇基础设施等有一定收益的项目建设。继续发行地方政府置换债券。
　　另一方面，稳健的货币政策要灵活适度。保持流动性合理充裕和社会融资总量适度增长，广义货币（M2）余额增长13%左右，与去年实际增速基本持平。一是疏通传导机制，降低实体经济融资成本，提高金融服务实体经济效率，推进解决融资难融资贵问题。二是拓宽债权、股权等融资渠道，发展项目收益债、高收益债券及可转换债券、可续期债券、永续票据等股债结合产品，提高直接融资比重。三是完善人民币汇率市场化形成机制，坚持有管理的浮动汇率制度，增强汇率双向浮动弹性，保持人民币汇率在合理均衡水平上的基本稳定，有效管控资本跨境异常流动。
　　在有效实施财政政策、货币政策的同时，统筹运用产业政策、区域政策、投资政策、消费政策、价格政策，增强政策之间的协调配合，加强逆周期调节，为经济发展和结构性改革营造稳定的宏观环境。
　　--产业政策要准。准确定位结构性改革方向，以供给创新促进需求增长，推动形成新的增长点。一是坚持创新驱动。强化创新激励和创新成果应用，实施科技成果转移转化行动和知识产权战略行动，改革科技成果产权制度，落实股权期权税收政策，完善分红奖励办法。发挥双创、“互联网+”集众智汇众力的乘数效应。二是注重激活存量。加快技术改造升级，修复现有产业和企业动力，提升产业层次，大力发展实体经济，推动中小企业创新转型发展。三是加快绿色发展。推进生态文明先行先试和制度建设，构建绿色产业体系和空间格局，引导形成绿色生产方式和生活方式。
　　--微观政策要活。完善市场环境，激发企业活力和消费者潜力。一是以解决放权不同步、不协调、不到位等问题为重点，深化行政审批制度改革，进一步放宽市场准入，提高监管能力和水平，为大众创业万众创新、企业投资经营和群众办事提供更加高效便捷的服务。二是保护各种所有制企业产权和合法利益，实施公平竞争审查制度，消除市场壁垒和地方保护，打破商品和要素自由流动限制，为各种所有制企业创新发展营造宽松的投资和市场经营环境。在项目核准、融资服务、财税政策、土地使用等方面，对民间投资与政府、国企投资一视同仁。三是通过创新供给、改善供给质量和效率，提高有效供给能力，促进供给与需求有效对接，满足不同消费群体多元化、个性化需求。
　　--改革政策要实。进一步提高改革方案质量，完善落实机制，把握好改革试点，确保改革见到实效，使群众有更多获得感。一是坚持问题导向。抓住主要矛盾，着力推进牵一发动全身的重要改革，依靠改革解决发展中的突出矛盾和问题。加快推进行政审批、投融资、价格、国企、财税金融、社会等领域改革。二是加强统筹协调。更加注重改革的系统性整体性协调性，做好重大和关键性改革的顶层设计，注重相关改革协同配套，形成改革合力。三是狠抓改革落地。解决好改革方案与实际相结合的问题、利益调整中的阻力问题、落实改革的责任担当问题，发挥基层首创精神，鼓励地方进行差别化探索，把改革落准落细落实。
　　--社会政策要托底。守住民生底线，发挥社会保障的稳定器作用。一方面，增加公共服务供给。坚持普惠性、保基本、均等化、可持续方向，保障群众基本生活，保障基本公共服务。另一方面，完善兜底性制度。分类扶持贫困家庭，增加失业救助、最低生活保障方面的财政资金，对无法依靠产业扶持和就业帮助脱贫的贫困家庭实施兜底保障政策。

　　**三、**2016年经济社会发展的主要任务
　　2016年，将紧紧围绕适应引领经济新常态、贯彻落实发展新理念，重点推进9方面工作，确保“十三五”开好局、起好步。
　　（一）推进供给侧结构性改革重点任务。调既有存量、改造提升传统动能，扩有效增量、培育发展新动能，提高供给结构的适应性和灵活性，增强持续增长动力。
　　一是积极稳妥化解过剩产能。更加注重运用市场机制、经济手段、法治办法，更加注重分类有序、因地施策、因业施策、因企施策，更加注重标本兼治、建立市场化产能调节长效机制。实行严格的环保、能耗、安全、技术标准，严控增量，严肃财经、信贷纪律，分类推进企业兼并重组、债务和解、破产重整乃至破产清算，积极稳妥处置长期亏损、失去清偿能力和市场竞争力的企业。加大财税、金融、土地等政策支持，促进钢铁、煤炭等行业脱困和转型升级，把职工安置作为化解产能过剩工作的重中之重，中央设立专项奖补资金，重点用于职工分流安置。
　　二是努力降低企业成本。开展降低实体经济企业成本行动，着力降低制度性交易成本、人工成本、税费负担、融资成本、用能用地成本、物流成本，研究降低社会保险费。精简涉企审批事项和办事程序，推进企业管理创新。合理把握最低工资标准提高幅度。进一步正税清费，实行涉企收费目录清单管理，全面推开营改增，加快资源税改革，研究精简归并“五险一金”，完善住房公积金制度，适当降低企业住房公积金缴存比例。深化利率市场化改革，发展股权融资，扩大债券发行规模，稳步降低企业综合融资成本。加快电力、热力、石油、天然气等体制机制改革。推进流通体制改革。
　　三是有序化解商品房库存。推进以满足新市民住房需求为主的住房制度改革，合理降低交易成本，鼓励农民工在中小城市购房，落实好首次购房优惠政策。培育发展住房租赁市场，鼓励自然人和各类机构投资者购买库存商品房，扩大租赁市场房源。提高棚户区改造以及其他房屋征收项目货币化安置比例，实施公租房货币化。用足用好住房公积金，把有能力有意愿的个体工商户和农民工逐步纳入住房公积金体制。
　　四是着力补齐短板。把脱贫攻坚同扩大有效供给、化解过剩产能有机结合起来，打好脱贫攻坚战。支持企业技术改造和设备更新。推动制造业高端化、智能化、绿色化、服务化，采用先进制造产业投资基金注资、股权投资、投资补助等方式，集中实施一批工程化、产业化示范项目，培育发展新兴产业。补齐软硬基础设施短板。以综合交通枢纽建设为核心，加快“最后一公里”水电气路、新一代信息基础设施、新能源汽车基础设施、城市地下管网、储气调峰设施、城际交通基础设施互联互通、城乡物流基础设施网络、生态保护和环境治理等建设。推动交通物流融合发展，提升综合效率和效益。全面加强教育事业，加快推进中西部教育发展，加强对农民工、职业农民、退役军人和下岗失业人员的培训。实施山水林田湖生态保护和修复工程，加大环境治理力度，改革环境治理基础制度，增强生态保护和环境治理能力，筑牢生态安全屏障。
　　五是防范化解金融等风险。加强经济运行监测预警和政策预研储备，创新和完善宏观调控方式，提高风险预测防范能力，保持经济运行在合理区间，确保发展的连续性和稳定性。加强金融宏观审慎管理制度建设，全方位监管系统重要性金融机构、金融控股公司和重要金融基础设施，健全前瞻性金融风险监测、评估和应对机制，完善股市、汇市、债市风险处置预案和金融机构退出机制，支持银行核销处置不良贷款，有序释放信用违约风险，规范各类融资行为，开展互联网金融风险专项整治，加大金融诈骗、非法集资打击力度，坚决守住不发生系统性区域性风险底线。着力缓解财政收支矛盾，完善地方政府债务限额管理、预算管理、风险预警和监督考核制度，完善全口径政府债务管理，改进地方政府债券发行办法。加强对实体经济的支持，防止企业“三角债”、资金链断裂放大蔓延。落实好援企稳岗、社保补贴、税费减免等政策，加大再就业支持力度。加强和创新社会治理，深入排查和调处化解社会矛盾，维护社会和谐稳定。
　　（二）用新思路新举措深挖内需潜力。有效发挥消费基础作用和投资关键作用，进一步夯实内需基础，保持经济运行在合理区间。
　　一是推动新消费引领新供给形成新动力。顺应居民消费结构升级趋势，着力推进供给创新，加快形成消费和供给良性互动、需求升级和产业升级协调共进的格局。2016年社会消费品零售总额预期增长11%左右。第一，千方百计提高居民消费能力。着力增加中低收入者收入。落实有利于科技成果转移转化的分配政策。健全促进农民增收机制，探索完善土地增值收益在国家和集体之间的分配机制。第二，大力培育和拓展消费热点。稳定住房和汽车等大宗消费。提高网速、降低网费，加快线上线下融合，支持可穿戴设备、智能家居、数字媒体等信息消费。培育新型服务消费，切实落实带薪年休假制度，发展乡村旅游、红色旅游、生态旅游、研学旅行，有序拓展“老幼”两端消费，扩大和引导文化消费。发展通用航空、邮轮游艇等时尚消费。倡导有利于节约资源、改善环境的绿色消费。充分释放农村消费潜力。第三，扩大有效供给释放潜在消费。鼓励支持地方和企业加大宽带乡村、中小城市信息基础设施、民用空间基础设施等建设力度，推动旅游基础设施和服务设施建设。支持发展城市文化娱乐综合体，推动社会力量举办的大型体育场馆免费或低费开放。丰富健康、家庭、养老、长期护理、文化创意等服务和产品供给。第四，改善消费环境。全面提高标准化水平，健全消费者权益保护机制。开展改善消费品供给专项行动，建立商品质量惩罚性赔偿制度。完善消费品进口关税等相关政策。实施食品安全战略，建立健全食品安全标准和追溯体系。提高药品质量。加强价格监管和反垄断执法，维护市场竞争秩序。
　　二是着力补短板、调结构、提高投资的有效性。牢牢抓住关键领域和薄弱环节，创新政府性资金使用方式，充分发挥中央预算内投资、专项建设基金“四两拨千斤”的引导带动作用，着力补短板，扩大有效投资。2016年全社会固定资产投资预期增长10.5%左右。第一，进一步优化用好预算内投资。中央预算内投资拟安排5000亿元，重点支持保障性安居工程、粮食水利、中西部铁路、科技创新、节能环保和生态建设、教育卫生文化等社会事业、老少边贫地区建设等。第二，充分发挥专项建设基金带动作用。按季度安排市场化方式筹措的专项建设基金，保持适度规模。积极推动社会资本跟进，发挥投贷结合效应。加大企业债券发行力度，鼓励发行城市地下综合管廊、停车场、战略性新兴产业、养老产业、配电网建设改造、双创孵化、绿色债券等企业债券创新品种。第三，组织编制三年滚动投资计划。切实加强政府投资项目储备，做到储备报批一批、开工建设一批、投产达标一批，形成接续不断、滚动实施的储备机制和良性循环。第四，着力激活社会投资。统筹安排中央预算内投资和专项建设基金，优先支持符合投向的政府和社会资本合作（PPP）项目建设。进一步放宽市场准入，拓宽社会资本投资渠道。加快出台政府投资条例、基础设施和公用事业特许经营条例、企业投资项目核准和备案管理条例。第五，加强关键领域薄弱环节重大工程建设。着力推进脱贫攻坚、棚户区改造、农村电网和城镇配电网改造、重大水利工程、铁路、公路、城市轨道交通、重点流域水污染治理和企业技术改造等“十三五”重点专项和重大项目建设。新开工20项重大水利工程。实施交通“一二三百”、西北风光电基地和军民融合重大战略工程。加强投资管理，加大项目监督稽察力度。

　　（三）加大重要领域和关键环节改革力度。抓紧推出一批具有重大牵引作用的改革举措，更加注重制度供给，不断释放市场活力。
　　一是推动简政放权放管结合优化服务向纵深发展。继续取消和下放一批行政审批事项，增强放权的协同性。全面公布地方政府权力和责任清单，开展国务院部门权责清单编制试点，在部分地区试行市场准入负面清单制度。推进综合执法和大数据监管，运用市场、信用、法治等手段协同监管。加快推进社会信用体系建设，拓展全国信用信息共享平台覆盖范围和服务功能，强化守信联合激励和失信联合惩戒机制。全面实施统一社会信用代码制度，推进“三证合一”、“一照一码”，开展“证照分离”试点。加快实施公共资源交易市场化改革，整合建立统一的公共资源交易平台。改进政府服务方式，推广“互联网+政务服务”，实现部门间数据共享，加快推进行政审批标准化建设。推动不动产统一登记制度在基层落地实施。
　　二是推进投融资体制改革。创新投资管理方式和投融资机制，进一步精简投资审批，减少、整合和规范报建审批事项，完善投资项目在线审批监管平台，建立新型企业投资项目并联核准制度。健全政银企社合作对接机制，推广特许经营等政府和社会资本合作（PPP）模式，推进社会资本参与重大工程建设运营。
　　三是深化价格机制改革。推动放开电力、石油天然气、交通运输等领域竞争性环节价格。扩大输配电价改革试点范围，完善环保电价政策。完善成品油价格形成机制。推进医疗服务价格改革。稳步推进农业水价综合改革。建立健全市场价格行为规则，加强市场价格行为监管，规范价格收费秩序。
　　四是打好国有企业改革攻坚战。加快改组组建国有资本投资、运营公司。推进国有企业混合所有制改革，在铁路、民航、电信、军工等重点领域，选择一批国有企业开展混合所有制改革试点示范。推进电力、石油天然气、盐业体制改革。出台进一步完善产权保护制度的意见。
　　五是加快财税体制改革。出台实施中央与地方事权和支出责任划分的指导意见。提高中央国有资本经营预算调入一般公共预算的比例。将建筑业、房地产业、金融业和生活服务业纳入营改增范围，实行不动产进项税抵扣，确保所有行业税负只减不增。全面实施资源税从价计征改革，推进消费税和环境保护税改革，逐步推进综合与分类相结合的个人所得税改革。
　　六是推进金融体制改革。丰富金融机构体系，深化国有商业银行和开发性、政策性金融机构改革，继续推进民营银行常态化发展。发展普惠金融，着力加强对中小微企业、农村特别是贫困地区金融服务。促进和规范新型金融业态健康发展。稳步推进人民币资本项目可兑换。推动多层次资本市场健康发展。完善金融监管制度。建立巨灾保险制度。
　　同时，继续深化民生领域和社会事业相关改革，推进教育、养老、文化、医药卫生体制改革，推动空域管理体制改革。
　　（四）实施创新驱动发展战略。贯彻创新发展理念，全面落实创新驱动发展战略纲要，深入开展大众创业、万众创新，深化科技体制改革，扩资源、拓空间、优环境，激发全社会特别是企业的创新创业创造活力和潜力。
　　一是增加创新资源供给。推进国家重大科技基础设施和国家实验室、产业创新中心建设，依托重大科技基础设施集聚区域建设综合性国家科学中心，实施一批新的重大科技项目和工程。系统推进全面创新改革试验，组织实施创新企业百强工程。
　　二是加快实施双创行动计划。推动建设若干双创示范基地，进一步打造众创众包众扶众筹支撑平台，大力发展创客空间和专业化众创空间。发展分享经济。鼓励地方和大企业设立创投基金、创业创新平台、科技创新中心等。重点扶持一批创业孵化、创业辅导、公共服务等平台。推进“互联网+”行动，实施大数据开放创新工程。建设高速、移动、安全、泛在的新一代信息基础设施，深入实施信息惠民工程，完善电信普遍服务机制。建设网上丝绸之路。继续办好双创活动周，再打造一批双创活动品牌。
　　三是大力发展战略性新兴产业。抓紧布局支撑战略性新兴产业发展的基础设施、公共资源库和全球创新网络，在高性能集成电路、新型显示、空间技术应用、通用航空、生物医药等战略性领域再布局一批重大项目和工程。设立国家战略性产业发展基金，开展融资风险补偿试点，推进战略性新兴产业区域集聚发展。
　　四是增强制造业核心竞争力。深入实施“中国制造2025”。围绕重点领域关键环节努力突破核心技术并实现产业化。加快发展制造业智能化技术装备。推动轨道交通、新能源汽车、医药、石化、煤化工、食品等产业健康发展。开展质量品牌提升行动，加强质量基础建设。
　　五是加快推动服务业优质高效发展。落实加快发展生产性服务业、生活性服务业的两个指导意见，进一步放宽服务业市场准入，营造公平竞争的环境，推动生产性服务业向专业化转变、向价值链高端延伸，推动生活性服务业加快向精细化和高品质提升。实施高技术服务业创新工程。启动新一轮国家服务业综合改革试点，及时推广可复制的经验和做法。
　　六是完善鼓励创新发展的政策和环境。落实研发费用加计扣除，完善高新技术企业、科技企业孵化器等税收优惠政策，建立新材料、关键零部件首批次应用保险费补偿机制。推进高校、科研院所改革，制定科研人员兼职或离岗创业、职务发明等相关规定，形成鼓励创业创新的收入分配机制。
　　（五）进一步夯实农业基础。确保农业农村投入力度不减弱、总量有增加，支持重大建设任务不打折扣。2016年粮食总产量稳定在5.5亿吨以上。
　　一是确保国家粮食安全。全面落实粮食安全省长责任制，完善中央和地方共同负责的粮食安全保障机制。探索划定粮食生产功能区和重要农产品生产保护区，保口粮、保耕地、保产能，保主产区特别是核心产区的粮食生产。全面完成永久基本农田划定并实行特殊保护，加强高标准农田、重要农产品生产基地、国家级育制种基地和区域性良种繁育基地、粮食仓储物流等建设。实施耕地质量保护提升行动。推进农作物良种补贴、粮食直补、农资综合补贴“三补合一”，健全以产粮大县、生猪调出大县等为核心的主产区利益补偿机制。改革完善粮食等重要农产品价格形成机制和收储制度。发展粮食产业经济。扩大农业政策性保险覆盖面，提高农业风险保障水平。
　　二是推进农村一二三产业融合发展。推动粮经饲统筹、农林牧渔结合、种养加一体，培育发展农村新型业态，拓展农业多种功能。实施“百县千乡万村”试点示范工程，完善农业产业链利益联结机制，让农民更多分享农村产业融合增值收益。实施农产品品牌提升行动，推进农业全产业链标准化、品牌化建设，严格生产管理、产品认证和证后监管。加强农产品、农资现代流通体系建设，健全农产品冷链物流网络，推动农产品线上营销与线下流通融合发展。深化农村集体产权、农垦、集体林权、国有林场、农田水利、供销社等改革。
　　三是推动农业可持续发展。以玉米为重点加快调整种养结构。编制实施耕地草原河湖休养生息规划。扩大农业面源污染综合治理、东北黑土地保护利用试点范围，启动地表水过度开发治理试点，加快耕地重金属污染、地下水超采治理。创建农业可持续发展试验示范区。
　　四是提高新农村建设水平。创新农村基础设施投融资体制机制。继续完善农村供电、供水、交通、通信设施和水利气象综合防灾减灾体系，启动实施农村饮水安全巩固提升工程，开展农村人居环境整治和农业废弃物资源化利用、无害化处置。把社会事业发展的重点放在农村和接纳农业转移人口较多的城镇，推动新型城镇化与新农村建设协调发展。
　　（六）促进城乡区域协调协同发展。贯彻协调发展理念，坚持区域协同、城乡一体，推动“三大战略”和“四大板块”形成叠加效应，构建核心带动和板块联动的区域开发新格局。
　　一是重点实施好“三大战略”。统筹推进“一带一路”建设。全面落实京津冀协同发展规划纲要，研究实施控增量、疏存量相关政策措施，启动一批北京非首都功能疏解重大项目，持续推进交通一体化、生态环境保护、产业升级转移3个重点领域率先突破，强化创新驱动试点示范，加快构建协同发展的体制机制。坚持生态优先、绿色发展，着力建设长江经济带“一道两廊三群”，把修复长江生态环境放在首要位置，大力构建绿色生态廊道，高起点高水平建设综合立体交通走廊，着力建设现代产业走廊，发挥长三角、长江中游、成渝三大城市群带动作用。
　　二是深入推进“四大板块”协调发展。支持宁夏、贵州等西部地区发展各具特色的内陆开放型经济，推进创新平台和创新体系建设。加大对中西部地区基础设施等薄弱环节的投资力度，加强生态保护和修复，培育壮大特色优势产业。实施新一轮东北地区等老工业基地振兴战略，培育形成以哈大经济带为主轴的东北地区城市群。出台促进中部地区崛起新十年规划，推动长江中游城市群、中原经济区、皖江城市带等重点经济区发展，推进淮河、汉江生态经济带建设，支持山西建设清洁能源基地。继续支持东部地区在改革创新、转型升级、提质增效上率先发展，发挥示范作用。深入推进海南国际旅游岛建设。大力发展海洋经济，推进海洋经济发展示范区建设。推进国家级新区、承接产业转移示范区等重要功能平台建设，鼓励各地先行先试，在体制机制改革、区域协同发展中当好排头兵。有序推广国家综合配套改革试验区成熟的改革经验。推进重点地区一体化发展，支持有条件的地区开展同城化探索。

　　三是以人为核心积极推进新型城镇化。制定实施1亿非城镇户籍人口在城镇落户方案。实施深入推进新型城镇化建设意见，加快户籍制度改革和居住证制度双落地，建立健全农业转移人口与建设用地、建设资金、转移支付“三挂钩”机制，鼓励引导农业转移人口在城镇落户。加大城镇存量建设用地盘活使用力度，积极推进农村土地征收、集体经营性建设用地入市、宅基地三项改革试点。制定全国城镇体系规划。持续推动新生中小城市、特色小城镇培育和新型城市建设，促进符合条件的开发区、陆路边境口岸地区完善城市综合功能。大力发展智慧城市、绿色城市和海绵城市。建设城市地下综合管廊，确保开工建设2000公里。加快京津冀、长三角、珠三角、成渝等城市群城际铁路建设。

　　（七）促进绿色循环低碳发展。贯彻绿色发展理念，推动能源生产和消费革命，提高发展的绿色化水平。
　　一是加快主体功能区建设。开展主体功能区规划修编，建立重点生态功能区产业准入负面清单制度，发布全国主体功能区规划拼图和农产品主产区、重点生态功能区目录，编制实施省级海洋主体功能区规划。开展省级空间规划试点。研究建立资源环境承载能力监测预警长效机制。
　　二是提高能源资源利用效率。出台2030年能源生产和消费革命战略，推动能源转型发展，实行能源消费总量和强度“双控”行动。提升可再生能源消纳能力，在解决好弃水、弃风、弃光问题的基础上，继续大力发展可再生能源。积极发展分布式能源，推进智能电网发展，推行节能低碳电力调度。加快发展节能环保绿色产业，实施绿色制造工程，支持绿色清洁生产、资源综合利用，实施节能环保重大工程。推进园区循环化改造、循环型产业体系构建、循环经济试点示范。实施建筑能效提升和绿色建筑全产业链发展计划。全面推进节水型社会建设。强化土地节约集约利用。
　　三是改善生态环境质量。深入实施大气、水污染防治行动计划，出台实施土壤污染防治行动计划，持续下大力气治理大气雾霾和水污染。严控新增煤炭产能和煤电新增规模，做好散煤清洁化利用和治理，实施电能替代，全面加快煤电升级改造和油品质量升级。推进环境污染第三方治理。全面实施新一轮退耕还林还草，加强国土绿化工程建设。稳步建立国家公园体制。继续推进京津风沙源、石漠化地区、退牧还草等重点区域生态及重点流域水环境综合治理。启动生物多样性保护重大工程。加强湖泊湿地生态保护，划定森林、草原、湿地、海洋等生态系统红线。健全生态保护补偿机制。推动战略和规划环评落地，严格环境准入。开展城市生态修复，恢复和提升城市生态功能。开展省以下环保机构监测监察执法垂直管理试点。
　　四是提升低碳发展水平。继续推进低碳试点示范。加快建设全国碳排放权交易市场，制定交易、监管等配套细则。建设性参与国际谈判，做好巴黎协定后续机制设计和推动落实，主动引导全球气候治理规则制定，继续加强气候变化“南南合作”。

　　（八）发展更高层次开放型经济。贯彻开放发展理念，优化对外开放区域布局，扩大对外投资合作，推进外贸优进优出，积极利用外资，培育国际竞争新优势，有效参与全球经济治理。
　　一是抓好“一带一路”建设战略落实。加强与沿线国家发展战略对接，稳步实施互联互通、能源资源合作等标志性工程，建立完善“一带一路”建设重大项目储备库，打造一批双多边合作样板。积极推进中蒙俄、中国－中南半岛等国际经济合作走廊建设。统筹推动中欧班列建设和沿线大通关合作，加快建设国际物流大通道。稳步构筑海上战略支点，畅通海上贸易通道。推进“一带一路”官方网站等信息平台建设。积极推动文化、中医药等走出去。深入推进沿边地区开发开放，提高边境经济合作区、跨境经济合作区发展水平，加强与周边国家的国际区域合作。
　　二是扩大国际产能和装备制造合作。围绕重点产业、重点国别，建立产能合作项目库。建立健全省区、牵头企业与重点国别对接机制，完善重大风险管理机制。加快推进双边产能合作机制建设，充分利用多边平台务实推进产能合作，积极与发达国家开展第三方市场产能合作，推动中国技术、中国装备、中国标准、中国服务走出去。完善财政金融支持政策，设立人民币海外合作基金、多双边产能合作基金，推动银企合作。2016年非金融类境外直接投资预期约1300亿美元，增长10%左右。
　　三是促进外贸稳增长、调结构。执行好出口退税政策。落实清理和规范进出口环节收费的各项政策措施，严格收费目录清单制度，加快国际贸易“单一窗口”和通关一体化建设，提高贸易便利化水平。促进加工贸易向中西部地区转移、向产业链中高端延伸。推动外贸新型商业模式发展，推进跨境电子商务综合试验区建设，扩大试点范围，支持建立一批“海外仓”和展示中心。推动货物贸易和服务贸易融合发展，大力促进金融、保险、物流、维修等服务贸易出口。实行更加积极的进口政策，支持先进技术设备、关键零部件进口，增加重要能源资源储备，适当增加消费品进口。深化多双边经贸合作，着力构建面向全球的高标准自贸区网络，积极商签区域全面经济伙伴关系协定，加快中日韩自贸区等谈判，推进中美、中欧投资协定谈判。
　　四是提升利用外资水平。进一步改善营商环境。推进外商投资负面清单管理模式改革，扩大金融、教育、养老、文化、电信、互联网、商贸物流等服务领域开放，进一步开放制造业，鼓励外资投向传统产业高端环节、战略性新兴产业和现代服务业。修订中西部外商投资优势产业目录，支持中西部地区承接外资产业转移。及时总结推广自贸试验区经验，进一步扩大试点范围。促进开发区创新发展，修订开发区审核公告目录。深化外商投资管理制度改革和外债管理改革，完善外商投资国家安全审查制度。2016年非金融类外商直接投资预期约1280亿美元。
　　（九）着力保障和改善民生。贯彻共享发展理念，把增进民生福祉放在突出位置，大力实施精准扶贫、精准脱贫，深化公共服务重点领域改革创新，全力保障基本公共服务供给。
　　一是全面推进精准脱贫攻坚。支持集中连片特困地区区域发展与脱贫攻坚，启动重点贫困革命老区振兴发展行动计划，加快革命老区开发建设步伐。加大对民族地区、边疆地区脱贫攻坚支持力度。着力改善贫困地区基础设施和基本公共服务，重点解决通路、通水、通电、通网。大力实施脱贫攻坚重点工程。继续支持新疆、西藏和四省藏区发展，加大中央支持和对口支援力度。

　　二是做好就业托底。统筹推进高校毕业生、农民工、就业困难人员等重点群体就业创业，做好产能过剩企业职工安置工作，继续实施高校毕业生就业促进计划和创业引领计划，健全公共就业创业服务体系，深入开展支持农民工等人员返乡创业试点。加强基层就业创业服务设施建设，强化就业援助，提供托底帮扶。发挥好战略性新兴产业、新业态、国家重大工程等对就业创业的拉动作用，培育壮大新的就业增长点。促进灵活就业和非正规就业。
　　三是强化社会保障关键环节建设。出台养老保险制度改革总体方案，继续提高退休人员基本养老金标准，推进职工基础养老金全国统筹。制定划转部分国有资本充实社保基金办法。全面推进城乡居民基本医疗保险制度整合，城乡居民基本医疗保险财政补助标准提高到每人每年420元，加快推进基本医疗保险异地就医结算，做好基本医疗保险与大病保险、医疗救助、疾病应急救助等政策衔接。统筹推进城乡社会救助体系建设，完善残疾人和困境儿童服务与保障体系。继续推进城镇保障性安居工程建设，改善中低收入群众和困难群众的住房条件。2016年，城镇棚户区住房改造600万套，农村危房改造314万户。
　　四是提高公共服务共建共享水平。建立国家基本公共服务清单，实施共享服务提升工程，完善义务教育、医疗卫生、公共文化、养老、公共体育、残疾人康复和托养、社会福利、基本殡葬等服务设施。扩大普惠性学前教育资源，规范农村学校布局调整，加快城乡义务教育公办学校标准化建设，加快推进高中阶段教育普及，实施支援中西部地区招生协作计划和面向贫困地区定向招生专项计划，畅通农村和贫困地区学子升学渠道，完善现代职业教育体系，实施职业教育产教融合工程。提升高校教学水平和创新能力，推动具备条件的普通本科高校向应用型转变。扩大各级各类学校和教育机构资源向城乡社区教育和老年教育开放。2016年，普通高等教育计划招生705万人，研究生招生81.8万人。加快全民健康保障工程建设，加强医疗服务体系薄弱环节，优化医疗卫生资源布局，大力培养全科医生、儿科医生，加快分级诊疗制度建设。实施全面两孩政策，完善妇幼健康服务体系和相关配套政策，促进人口均衡发展。积极发展中医药、民族医药事业。推进养老服务业综合改革试点。完善流动人口融入城镇的基本公共服务政策。加强社会主义精神文明建设。加快文化改革发展。繁荣发展哲学社会科学。发展文学艺术、新闻出版、广播影视、档案等事业。建设中国特色新型智库。加快足球、冰雪等各类体育运动发展。
　　深化内地与香港、澳门的投资经贸合作，落实内地与港澳关于建立更紧密经贸关系安排（CEPA）框架下的服务贸易协议，加大内地对港澳开放力度，推动CEPA全面升级。支持港澳参与“一带一路”建设。加快前海、南沙、横琴等粤港澳合作平台建设，深化泛珠三角等区域合作。进一步支持澳门建设中国与葡语国家商贸服务平台，促进澳门经济适度多元发展。继续推进海峡两岸经贸关系发展，增进两岸同胞福祉。
　　各位代表，做好“十三五”开局之年的经济社会发展工作，任务艰巨，意义重大。我们要更加紧密地团结在以习近平同志为总书记的党中央周围，高举中国特色社会主义伟大旗帜，以邓小平理论、“三个代表”重要思想、科学发展观为指导，深入贯彻习近平总书记系列重要讲话精神，自觉接受全国人大的监督，虚心听取全国政协的意见和建议，齐心协力、奋发有为，攻坚克难、开拓创新，为实现“十三五”良好开局、保持经济社会平稳健康发展而努力奋斗！

第十二届全国人民代表大会财政经济委员会关于2015年国民经济和
　　社会发展计划执行情况与2016年国民经济和社会发展计划草案的审查结果报告
　　（2016年3月13日第十二届全国人民代表大会第四次会议主席团第二次会议通过）

十二届全国人大四次会议主席团：
　　第十二届全国人民代表大会第四次会议审查了国务院提出的《关于2015年国民经济和社会发展计划执行情况与2016年国民经济和社会发展计划草案的报告》以及2016年国民经济和社会发展计划草案。全国人民代表大会财政经济委员会在对计划报告和计划草案初步审查的基础上，根据各代表团和有关专门委员会的审查意见，又作了进一步审查。国务院根据审查意见对计划报告作了修改。现将审查结果报告如下。
　　财政经济委员会认为，2015年国民经济和社会发展计划执行情况总体是好的。面对错综复杂的国际环境和艰巨繁重的国内改革发展稳定任务，国务院和地方各级人民政府认真落实党中央决策部署和十二届全国人大三次会议提出的各项要求，主动适应经济发展新常态，坚持稳中求进工作总基调，妥善应对重大风险挑战，完成了全年主要目标任务，经济社会发展和改革开放事业取得重大进展。国民经济运行总体平稳，稳中有进，稳中有好，经济结构继续优化，改革开放向纵深迈进，民生不断得到改善，社会大局总体稳定，成绩来之不易。同时也要看到，经济社会发展仍存在不少困难和挑战。主要是：部分指标没有达到预期，经济下行压力较大；有效需求乏力和有效供给不足并存，创新能力不强，经济结构调整和转型升级任务艰巨；企业成本上升，生产经营困难加大，部分企业裁员和隐性失业值得关注；国内主要农产品生产成本高、库存积压，农民增收乏力等问题突出，农业发展方式亟待转变；财政金融领域存在风险隐患，防范发生系统性区域性风险压力增加；生态环境保护、安全生产形势依然严峻，民生保障以及社会管理等方面仍存在不少矛盾和问题。对此，要采取措施认真加以解决。
　　财政经济委员会认为，国务院提出的2016年计划报告与计划草案，符合《[中共中央关于制定国民经济和社会发展第十三个五年规划的建议](https://www.pkulaw.com/chl/e69521f41400e9d7bdfb.html)》和中央经济工作会议精神，符合我国经济社会发展实际，指导思想明确，重点突出，主要目标和工作安排总体可行，建议批准国务院提出的《关于2015年国民经济和社会发展计划执行情况与2016年国民经济和社会发展计划草案的报告》，批准2016年国民经济和社会发展计划草案。
　　财政经济委员会认为，2016年是全面建成小康社会决胜阶段的开局之年，也是推进结构性改革的攻坚之年。在计划执行过程中，要全面落实党的十八大和十八届三中、四中、五中全会以及中央经济工作会议精神，深入学习贯彻习近平总书记系列重要讲话精神，按照“五位一体”总体布局和“四个全面”战略布局，牢固树立和贯彻落实创新、协调、绿色、开放、共享的新发展理念，不断增强适应新常态、把握新常态、引领新常态的自觉性，坚持稳中求进工作总基调，实行宏观政策要稳、产业政策要准、微观政策要活、改革政策要实、社会政策要托底的总体思路，着力抓好去产能、去库存、去杠杆、降成本、补短板工作，保持经济运行在合理区间，努力实现“十三五”时期经济社会发展良好开局。为此，财政经济委员会提出以下建议：
　　（一）积极推进供给侧结构性改革。在适度扩大总需求和调整需求结构的同时，把改善供给结构、提高资源配置效率作为主攻方向。加快技术、产品、业态等创新，不断创造新供给、提高供给质量。继续推进产业结构优化升级，支持企业技术改造和设备更新，大力培育新兴产业，积极发展健康、文化、养老、旅游等服务业。加强政策引导和支持，多措并举化解过剩产能，积极稳妥化解房地产库存。深入实施创新驱动发展战略，发挥企业在创新中的主体作用，激发微观主体活力，激励大众创业、万众创新。全面推进各领域改革，加大财税、金融、国企、价格、社保等重要领域和关键环节改革力度，进一步转变政府职能，简政放权、放管结合、优化服务，不断完善公平竞争、优胜劣汰的市场环境和机制。完善营商环境和开放布局，加快服务贸易发展，培育对外经济新优势。
　　（二）切实加大对实体经济支持力度。继续实施积极的财政政策和稳健的货币政策，统筹运用多种政策手段支持实体经济发展。财政政策要加大力度，进一步落实正税清费、减免税费等政策。采取综合措施，降低企业交易、人工、物流、财务、用能等成本，减轻企业社会保险费负担，清理规范中介服务收费。合理把握最低工资标准提高幅度。优化投资结构，提高投资效益。货币政策要灵活适度，保持流动性合理充裕，稳定资本市场，扩大直接融资比重，降低实体经济融资成本，推动解决融资难融资贵问题，提高金融服务实体经济效率。保护各种所有制企业的合法权益，鼓励和支持各种所有制企业创新发展，提高企业投资信心，改善企业市场预期。
　　（三）进一步加快转变农业发展方式。坚持以市场需求为导向，调整优化农业结构，推进农村一二三产业融合发展，不断提高农业综合生产能力，促进农民持续增收。依法推进土地经营权有序流转，加快发展多种形式适度规模经营。深化农村集体产权制度、农垦和供销社综合改革。改革农产品价格形成机制，完善粮食等重要农产品收储制度。推动农药、化肥、农膜减量使用，加强农业面源污染防治，推广农业标准化生产，健全从农田到餐桌的农产品质量和食品安全监管体系。扎实推进新农村建设，继续改善农民生产生活条件。加快农业转移人口市民化，建立健全财政转移支付同农业转移人口市民化挂钩机制，促进新型城镇化与农业现代化相辅相成，协调发展。
　　（四）大力推进资源节约和生态环境保护。制定出台生态文明目标评价考核办法，更加注重促进形成绿色生产方式和生活方式。强化约束性指标管理，实行能源和水资源消耗、建设用地等总量和强度双控行动，培育和发展排污权、碳排放权交易市场。积极发展和利用风能、太阳能、生物质能等清洁能源，降低煤炭在能源消费中的比重。提高煤炭利用集中度和燃煤技术标准，推广煤炭清洁高效利用技术，提升燃油品质，下大力气治理大气雾霾。大力发展节能环保绿色产业，开展传统制造业绿色改造，支持绿色清洁生产，加快发展循环经济。全面推进城镇污水处理设施建设与改造，加大农村环境治理力度，加强流域水环境综合治理。严格执行新环保法，努力提升环保监测水平和监管能力。
　　（五）继续发展社会事业和改善民生。推动教育公平发展和质量提升，统一城乡义务教育经费保障机制，加大公共教育投入向中西部和边远、贫困地区的倾斜力度。推动科技、文化、卫生、体育等社会事业改革发展。实施更加积极的就业政策，鼓励以创业带动就业，抓好技能培训和再就业，妥善应对部分职工下岗失业、收入降低等问题。制定划转部分国有资本充实社会保障基金办法。推进职工基础养老金全国统筹，完善基本养老金合理调整机制。抓紧研究制定应对人口老龄化措施和生育二孩的配套政策。加强基层医疗卫生服务体系建设，加快推进基本医保全国联网和异地就医结算，实现大病保险全覆盖，有效减轻大病患者就医负担。落实临时救助制度，完善最低生活保障、特困人员供养、残疾人保障等兜底性制度。支持慈善事业发展。推动精准扶贫和脱贫，确保完成贫困人口脱贫的年度任务。
　　（六）有效防范化解潜在风险。密切关注国内外形势的变化，高度重视经济下行带来的潜在风险，处理好“去产能、去库存、去杠杆”与防风险的关系。做好地方政府存量债务置换工作，高度关注一些地区新出现的债务风险苗头，完善全口径政府债务管理，推动地方政府融资平台市场化转型，有效化解地方政府债务风险。依法处置信用违约，加快商业银行不良贷款核销和处置进度。规范各类融资行为，严厉打击非法集资活动，开展互联网金融风险专项整治，妥善处理风险案件。抓紧研究提出金融监管体制改革方案，强化综合监管和功能监管，落实监管责任。增强忧患意识，加强风险监测预警，准备好应对之策和处置预案，坚决守住不发生系统性区域性风险的底线。
　　以上报告，请予审议。

第十二届全国人民代表大会财政经济委员会
　　2016年3月13日

© 北大法宝：（[www.pkulaw.com](https://www.pkulaw.com/)) 专业提供法律信息、法学知识和法律软件领域各类解决方案。北大法宝为您提供丰富的参考资料，正式引用法规条文时请与标准文本核对。
欢迎查看所有[产品和服务](https://www.pkulaw.com/helps/2.html)。法宝快讯：[如何快速找到您需要的检索结果？](https://www.pkulaw.com/helps/70.html)[法宝](https://www.pkulaw.com/helps/69.html)V6有何新特色？

扫描二维码阅读原文

**原文链接：**<https://www.pkulaw.com/chl/40530ca3120e490ebdfb.html>
